# Blood and Serum Se and Zn Levels and 10-Year Survival of Patients after a Diagnosis of Kidney Cancer

**DOI:** 10.3390/biomedicines12081775

**Published:** 2024-08-06

**Authors:** Elżbieta Złowocka-Perłowska, Piotr Baszuk, Wojciech Marciniak, Róża Derkacz, Aleksandra Tołoczko-Grabarek, Marcin Słojewski, Artur Lemiński, Michał Soczawa, Milena Matuszczak, Adam Kiljańczyk, Rodney J. Scott, Jan Lubiński

**Affiliations:** 1 Department of Genetics and Pathology, International Hereditary Cancer Center, Pomeranian Medical University, 70-204 Szczecin, Poland; piotr.baszuk@pum.edu.pl (P.B.); aleksandra.toloczko.grabarek@pum.edu.pl (A.T.-G.); matuszczakmilena@gmail.com (M.M.); adam.kiljanczyk@pum.edu.pl (A.K.);; 2 Read-Gene, Grzepnica, ul. Alabastrowa 8, 72-003 Dobra, Poland; wojciech.marciniak@read-gene.com (W.M.); roza.derkacz@read-gene.com (R.D.); 3 Department of Urology and Oncological Urology Clinic, Pomeranian Medical University, 70-204 Szczecin, Poland; marcin.slojewski@pum.edu.pl (M.S.); michal.soczawa@pum.edu.pl (M.S.); 4 Department of Biochemical Research, Pomeranian Medical University, 70-204 Szczecin, Poland; artur.leminski@pum.edu.pl; 5 School of Biomedical Sciences and Pharmacy, Centre for Information-Based Medicine, Hunter Medical Research Institute, University of Newcastle, Newcastle, NSW 2305, Australia; rodney.scott@newcastle.edu.au; 6 Division of Molecular Medicine, Pathology North, NSW Pathology, Newcastle, NSW 2305, Australia

**Keywords:** kidney cancer, survival, selenium, zinc

## Abstract

The aim of the project was to evaluate the association between selenium (Se) and zinc (Zn) levels in blood and serum and kidney cancer mortality. In a prospective group of 284 consecutive, unselected patients with kidney cancer, we evaluated their 10-year survival rate in relation to the levels of Se and Zn in their blood and serum. Micronutrient levels were measured using an inductively coupled plasma mass spectrometer. Patients were divided into quartiles based on the distribution of Se and Zn levels arranged in increasing order. The following variables were taken into account in the multivariable models: age at diagnosis, gender, smoking, type of surgery and histopathological examination results. We observed a statistically significant association of all-cause mortality when subgroups with low blood selenium levels were compared to patients with high selenium levels (HR = 7.74; *p* < 0.001). We found, in addition, that this correlation was much stronger when only men were assessed (HR = 11.6; *p* < 0.001). We did not find a statistically significant association for zinc alone. When we combined selenium and zinc levels (SeQI-ZnQI vs. SeQIV-ZnQIV), we observed the hazard ratio for kidney cancer death to be 12.4; *p* = 0.016. For patients in the highest quartile of blood zinc/selenium ratio, compared to those in the lowest, the HR was 2.53; *p* = 0.008. Our study suggests that selenium levels, combined selenium and zinc levels (SeQI-ZnQI vs. SeQIV-ZnQIV) and zinc-to-selenium ratio (Zn/Se) are attractive targets for clinical trials aimed at improving the survival of kidney cancer patients.

## 1. Introduction

Malignant kidney tumors account for 2–3% of the cancer burden. Among kidney cancer patients, the 5-year survival rate is 55% in men and 62% in women [1]. According to the American Cancer Society, the 10-year survival rate after a diagnosis of renal cell carcinoma is around 50% [2]. Becker F et al. observed that the 10-year survival rate for kidney cancer was 95.8% and 84.4% in NSS (partial resection of the organ with the tumor) patients and RN (radical nephrectomy) patients, respectively [3]. In our observation, the 10-year survival rate of patients after a diagnosis of kidney cancer is 72%. In the other cancers, the 10-year survival rate is about 86% for breast, over 65% for papillary thyroid cancer, about 50–75% for prostate and 15–17% for ovarian cancer [4,5,6,7,8,9].

The etiology of renal cancer is multifactorial. It includes age, obesity, viral hepatitis, type-2 diabetes, hypertension, exposure to cadmium, trichloroethylene, smoking and chronic kidney disease [10,11]. Approximately 5% of kidney cancers result from inherited predispositions [12].

Identifying biomarkers for cancer risk and subsequent prognostication is critically important in ensuring the best outcomes for patients. There is strong evidence of an association between the expression of selenium-containing proteins in serum and cancer risk and tumor aggressiveness [13,14,15]. Similarly, zinc has profound anti-cancer effects [16]. Lubiński et al. (2017) discovered that in a group of women with breast cancer who had smoked cigarettes in the past, there was an increased correlation between serum selenium concentration and survival [17]. The relationship between the number of cigarettes smoked and the risk of renal cell carcinoma is also well-established [18]. Recent work conducted by our research team has shown an association between high levels of selenium and zinc and reduced mortality among patients with breast, prostate, lung and laryngeal cancers [19]. We also reported a similar correlation between serum selenium levels and the risk of death in pancreatic cancer and malignant melanoma patients [17,20,21,22,23,24,25,26]. Similar findings have been reported by Sandsveden et al. (2020) and Psathakis et al. (1998) for patients with breast and colorectal cancers [27,28]. Several additional studies have shown that zinc deficiency in patients with breast, gastric, colorectal, lung and prostate cancers and leukemia correlates with disease progression and shorter survival [29,30]. 

Therefore, both Se and Zn are potentially good prognostic candidates for assessing the survival of kidney cancer patients. Our goal is to determine the association between selenium (Se) and zinc (Zn) levels in blood and serum and kidney cancer mortality.

To date, apart from one small study on the survival of patients with kidney cancer and the correlation between selenium levels and outcome (a study involving only 41 patients), there are no reports on the correlation between the survival of patients with kidney cancer and selenium and zinc levels in the blood and serum [31]. 

## 2. Materials and Methods

### 2.1. Study Group

Our study includes a series of 284 unselected kidney cancer patients diagnosed at the Urology and Oncological Urology Clinic, University Hospital in Szczecin, between 2014 and 2017. All patients signed written informed consent to obtain blood/serum samples for research purposes. The blood/serum samples were taken from patients at the time of diagnosis but before treatment and were stored in a research biobank. Patients were requested to fast before the blood/serum samples were collected. The patients were unselected for age, sex, smoking status, clinical characteristics (type of surgery and histological features), and if it occurred, the cause of death. Detailed information on the kidney cancer study population is shown in Table 1. The vital status and the date of death were obtained from the Polish Ministry of Internal Affairs and Administration in November 2023. The study was approved by the Ethics Committee of Pomeranian Medical University in Szczecin under number KB-006/07/22.

### 2.2. Sample Collection and Storage

Two 10 cm3 peripheral blood samples were taken from each patient. One blood sample was collected in an EDTA (ethylenediaminetetraacetic acid) vacuum tube and stored at −80 °C until the day of analysis. The second one was transferred to a tube with a clot activator, and it was incubated at room temperature for at least 30 min and then centrifuged at 1300× *g* for 12 min. The serum was stored at −80 °C. The blood sample and sera were thawed and then mixed by vortexing. The sera samples were centrifuged at 5000× *g* for 5 min on the day of analysis.

### 2.3. Measurement Methodology

The micronutrient levels of selenium (Se) and zinc (Zn) in the blood and serum were investigated using ICP-MS (an inductively coupled plasma mass spectrometer) (ELAN DRC-e, Perkin Elmer, Waltham, MA, USA). The spectrometer was tuned prior to each analysis, and external calibration was used to calibrate it using the matrix-matched calibration technique. The calibration standards for the determination of Se and Zn (1; 2; 3; 4; 5; 10; 50; 75; 100; 120; 150; and 170 µg/L) were prepared fresh daily by diluting 10 µg/mL Multi-Element Calibration Standard 3 (Perkin Elmer, USA) with a blank reagent. The reaction gas was oxygen. The correlation coefficients for the calibration curves were always greater than 0.999. The analysis protocol involved diluting the serum 40-fold in a blank reagent consisting of high-purity water (>18 MΩ), TMAH, Triton X-100, ethanol and EDTA. The LOD and LOQ for different materials are provided in Table 2, and technical details are available on request. 

### 2.4. Quality Control

The accuracy and precision of all measurements were tested using certified reference material (CRM), Clincheck Plasmonorm Blood Trace Elements Level 1 (Recipe, Munich, Germany), Clincheck Plasmonorm Serum Trace Elements Level 1 (Recipe, Germany) and Seronorm Serum Trace Elements Level 1 (Sero, Hvalstad, Norway). Technical details, plasma operating settings, and mass spectrometer acquisition parameters can be provided upon request. The testing laboratory participates in independent external quality assessment schemes for determining trace elements in blood: QMEQAS (Quebec Multielement External Quality Assessment Scheme) organized by the Institut National de Santé Publique du Québec. Comparisons between the obtained and certified values of concentrations of selenium and zinc are provided in Table 3.

### 2.5. Statistical Analysis

In order to estimate the relationship between blood and serum selenium and zinc levels and kidney cancer survival, univariable and multivariable Cox regression models were used. The blood and serum levels of selenium and zinc were assigned to one of four subgroups (quartiles) in relation to the concentration of each of them and then arranged in ascending order. The quartiles with the lowest number of deaths observed were chosen as a reference range for each of the analyzed elements. An observation time ≥10 years were treated as a 10-year follow up in all calculations. In the multivariable models, the following variables were taken into account: age of diagnosis (≤60/>60), sex (female/male), smoking status (no/yes-former smoker), type of surgery (nephrectomy/tumorectomy), G (I-IV Fuhrman Grade system for nuclear grade: GI—nucleoli absent, inconspicuous at 400×; GII—nucleoli eosinophilic visible at 400×; GIII—nucleoli eosinophilic prominent at 100×; and GIV—nuclei pleomorphic, giant, rhabdoid and sarcomatoid), and histopathological results (clear cell/papillary-chromophobe). Probability results with a *p*-value ≤ 0.05 were considered statistically significant. Kaplan–Meier curves graphically illustrated the relationship between quartiles of analyzed elements and survival in kidney cancer patients. The calculations were performed using the R environment (R Foundation for Statistical Computing, Vienna, Austria, 2023; R version: 4.3.2).

## 3. Results

The study included 118 women and 166 men with kidney cancer. The mean age of diagnosis for all participants was 59 years. A total of 86% of patients were diagnosed with clear cell carcinoma, and 29% were diagnosed with Fuhrman grade GIII/IV. A total of 67% of cases had a history of smoking. Death from cancer was reported in 71% of patients with kidney cancer.

**Figure 1:**
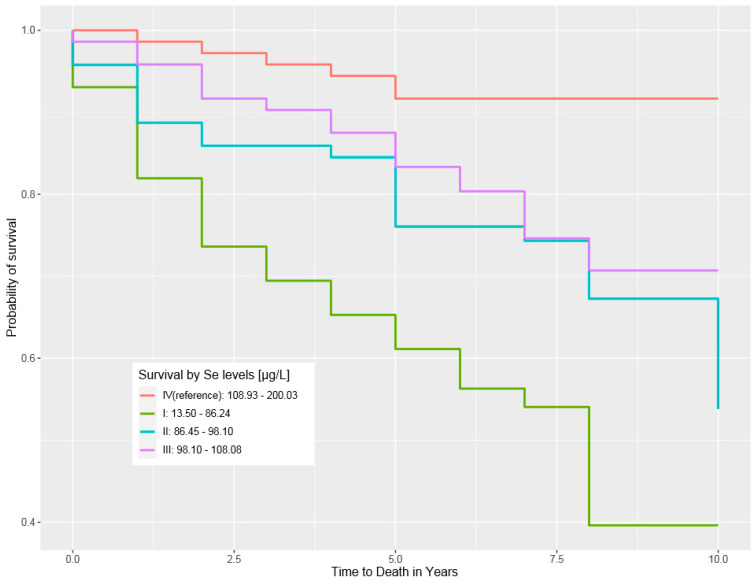
Ten–year survival by Se blood level.

The correlations between levels of Se in blood/serum and ten-year survival are presented in Table 4 and Table 5 and in Figure 1.

### 3.1. Selenium

The association between blood selenium levels and the death of patients with kidney cancer was highly significant and is shown in Table 4. We observed that the subgroup with low selenium concentrations had a significantly higher all-cause mortality rate compared to patients with higher selenium levels, HR = 7.74; *p* < 0.001. The association with all-cause mortality rates was more highly significant for men compared to women. The men with the lowest blood selenium levels had lower survival rates compared to those with high levels, HR = 11.6; *p* < 0.001 (Supplementary Material Tables S1–S4). There were no significant differences in selenium levels for all-cause mortality in relation to women, HR = 1.52; *p* = 0.5. When we combined selenium and zinc levels (SeQI-ZnQI vs. SeQIV-ZnQIV), the HR for all-cause mortality was 12.4; *p* = 0.016 for the multivariable model. The multivariate hazard ratio (HR) for the zinc-to-selenium ratio (Zn/Se ratio) in blood was 2.53; *p* = 0.008. 

The HRs were lower for serum levels and all-cause mortality. The results are as follows: 3.97; *p* = 0.001 for quartile I vs. IV, 3.54; *p* = 0.003 for quartile II vs. IV and 1.80; *p* = 0.2 for quartile III vs. IV. Similar findings were observed for all-cause mortality among men, HR = 4.95; *p* = 0.002 (Supplementary Material Tables S5–S8). There were no significant differences in serum selenium levels for all-cause mortality in relation to women, HR = 0.71; *p* = 0.6. In serum from patients who were in both the lowest Se quartile and the lowest Zn quartile (SeQI-ZnQI), the HR was 3.11; *p* = 0.049. For patients in the highest quartile of serum zinc/selenium ratio compared to those in the lowest, the HR was 2.08; *p* = 0.03. 

### 3.2. Zinc

The correlation between Zn levels in the blood or serum and deaths from kidney cancer showed similar tendencies to selenium; however, during the multivariable Cox regression analysis, statistical significance was not achieved (Supplementary Material Tables S9 and S10).

## 4. Discussion

This prospective study of 284 kidney cancer patients showed the correlation between selenium blood/serum levels and 10-year survival.

For blood samples, the HRs were higher for selenium levels and all-cause mortality rates compared to serum samples. We observed that high blood/serum selenium levels resulted in lower all-cause mortality rates among patients with kidney cancer. This was more significant (7-fold greater) in men compared to women. There was a significant correlation found when we combined selenium and zinc levels (SeQI-ZnQI vs. SeQIV-ZnQIV). Selenium and zinc are essential micronutrients. Selenium plays a role in mitigating oxidative stress, protecting DNA from attacks by reactive oxygen species (ROS), detoxifying carcinogens, inhibiting angiogenesis, the invasion of cancer cells, improving immunity by reducing the formation of DNA adducts and chromosome breaks [32,33,34,35]. Selenium inhibits the phase I enzymes of the cytochrome P450 system. These enzymes convert carcinogens into reactive adducts that attack DNA. It increases the activities of DNA repair enzymes, including DNA glycosylase, and repair pathways, including BRCA and p53, protecting against DNA damage [36]. Zinc is involved in oxidative stress and immune response as a neurotransmitter; it participates in growth retardation, cell division, proliferation, and apoptosis, and it contributes to cellular homeostasis [37,38,39]. Two protein families of zinc transporters, ZnT (responsible for the export of zinc from the cytosol) and ZIP (responsible for the export of zinc to the cytosol), regulate Zn metabolism, and they are responsible for the control of zinc homeostasis [40]. In some cancers, Zn homeostasis is disturbed, and the expression of ZnT and ZIP is abnormal. This can contribute to a loss of essential physiological functions and lead to structural abnormalities in cells or the development of cardiovascular diseases, diabetes and cancers [37,41,42]. Zinc transport proteins play different roles in different types of cancer. In pancreatic adenocarcinoma, overexpression of the ZIP4 protein increases cell proliferation, leading to tumor progression [43]. In prostate cancers, the expression of ZIP1 and ZIP4 is lowered, and they can act as tumor suppressors [44]. In turn, in renal cell carcinomas grades III-IV, a high expression of ZIP10 can suggest the aggressiveness of renal cancer [45]. In breast tumors, there are different levels of Zn for different breast cancer subtypes [46]. Dysregulation of Zn transport proteins could contribute to cancer progression, invasion and metastasis. The interactions that may occur between selenium and zinc are important. Depending on the level of zinc released, selenium has an antioxidant or pro-oxidant effect [47]. By oxidizing zinc fingers and transcription regulators of DNA repair genes, selenium can disrupt and inhibit the DNA repair process. If zinc homeostasis is disturbed, depending on selenium concentration, the metallothionein antioxidant system will be disrupted, resulting in oxidative DNA damage and cancer. Therefore, it is important to control the balance of these two elements [48]. The relationship between overall survival or cancer progression and blood/serum selenium and zinc levels is poorly described in the literature. We observed a greater association between patients in the highest quartile of the zinc/selenium ratio and those in the lowest. We observed that the subgroups with low selenium levels had significantly higher all-cause mortality rates compared to patients with higher selenium levels. By analyzing the zinc correlation between blood/serum zinc levels and deaths in kidney cancer patients, we observed a trend towards higher all-cause mortality only in the patients with the lowest zinc levels. 

The effect of specific micronutrients on the survival of patients with different types of malignancies, such as cancers of the breast, prostate, lung, larynx, pancreas, stomach, colon, renal, leukemia and malignant melanoma, has been reported previously [17,19,20,21,22,23,24,25,26,27,28,29,30,31], suggesting that our findings in kidney cancer indicate a generalized effect of Se/Zn levels on cancer outcomes.

We reported that women with breast cancers and lower serum selenium levels have a worse prognosis and survival than women with higher levels despite the lack of association between serum selenium levels and tumor characteristics or treatment [17]. A similar observation was made by Sandsveden et al. They noted that lower mortality in breast cancer is associated with higher serum selenium levels and is an independent prognostic factor [28]. Several studies have been conducted showing a correlation between serum selenium levels and mortality in laryngeal, lung, prostate, kidney and pancreatic cancer. Serum selenium levels above 70 μg/L showed much better survival in patients with the aforementioned cancers [22,23,24,26,31]. However, studies on kidney and pancreatic cancers have been performed only on small patient series (between 41 and 100 patients) [31]. Rogoża-Janiszewska et al. (2021) observed in melanoma patients that serum selenium levels below 76.44 µg/L were associated with increased mortality within 10 years of diagnosis [21]. Pietrzak et al. (2024) revealed that combined Se and Zn levels had a similar effect on survival in patients with prostate cancer. The patients with the lowest Se and Zn levels had a 21-fold lower probability of 5-year survival compared to those with the highest levels of Se and Zn [26]. In the aforementioned studies, the evidence suggests that a low serum Se level measured at the time of cancer diagnosis is a strong predictor of increased risk of death. A low Se level may also be a prognostic indicator of survival in kidney cancer patients. In 1974, Schrauzer et al. observed that the mortality rate of breast cancer patients was lower in selenium-rich areas of the US than in regions deficient in this element [49]. A meta-analysis that included 1910 cancer patients and 17,339 control patients showed that selenium supplementation was associated with reduced cancer mortality [50]. 

Gelbard et al. (2022) showed that zinc deficiency in patients with breast, stomach, colon, leukemia and lung cancer correlates with disease progression and worse survival rates [29]. Similar observations were made by our group in patients diagnosed with breast, prostate, laryngeal and lung cancer [19,25]. Similarly, a study by Epstein et al. (2011) found that higher Zn levels in prostate cancer patients correlated with better survival [30]. In contrast, Fang et al. (2019) and Bengtsson et al. (2022) found no significant association between serum zinc levels and survival in hepatocellular carcinoma and breast cancer [51,52]. In a subsequent report, Bengtsson et al. showed a tendency between lower serum zinc levels and worse breast cancer survival. Zinc deficiency correlates with poorer survival among cancer patients [29]. Our study also showed only a non-significant tendency between zinc levels and the survival of kidney cancer patients.

Herein, for the first time, we could see a very clear difference in the effects of selenium depending on sex. Higher selenium levels appeared more important for men. Validation of these findings warrants further investigation in much larger patient populations. In recent years, there has been growing interest in exogenous factors, including diet, that influence cancer mortality. Our data support the use of diets that potentially optimize micro-elements levels to improve treatment outcomes for cancer patients.

Our study has several limitations. First, this study was conducted at a single medical center. Second, the number of patients in our cohort is relatively low. In the future, it will be important to conduct multi-center validation studies. The strength of our study is that we are the first to examine the effect of selenium on the survival of kidney cancer patients, and we examined blood and serum collected from the same patients at the time of diagnosis but before treatment. In addition, this study is a good start to initiate collaboration with other researchers around the world to validate our results on the role of microelements in improving patient outcomes.

## 5. Conclusions

In conclusion, the current study showed that high levels of selenium in blood/serum, combined selenium and zinc levels (SeQI-ZnQI vs. SeQIV-ZnQIV) and zinc-to-selenium ratio predispose kidney cancer patients to better survival rates. The patients with the lowest selenium and zinc levels had a significantly higher all-cause mortality rate compared to patients with high selenium and zinc levels.

## 6. Patents

Based on the results presented in the following paper, a patent application has been submitted to the Patent Office of the Republic of Poland (P. 448864).

## Figures and Tables

**Table 1:** Characteristics of the patients in the study.

Variables	Overall N = 284	Alive N = 204	Dead N = 80
Age of diagnosis (mean)			
≤60 (50.12)	121 (43%)	98 (48%)	23 (29%)
>61 (67.66)	163 (57%)	106 (52%)	57 (71%)
Sex			
Female	118 (42%)	91 (45%)	27 (34%)
Male	166 (58%)	113 (55%)	53 (66%)
Smoking status			
No	95 (33%)	77 (38%)	18 (23%)
Yes/Former smoker	189 (67%)	127 (62%)	62 (78%)
Kind of operation			
Nephrectomy	126 (44%)	84 (41%)	42 (53%)
Tumorectomy	158 (56%)	120 (59%)	38 (48%)
Histological features			
* GI	75 (26%)	64 (31%)	11 (14%)
GII	125 (44%)	96 (47%)	29 (36%)
GIII	63 (22%)	39 (19%)	24 (30%)
GIV	21 (7.4%)	5 (2.5%)	16 (20%)
Clear cell carcinoma	245 (86%)	169 (83%)	76 (95%)
Papillary/Chromophobe	39 (14%)	35 (17%)	4 (5.0%)
Death due to cancer			
No	-	-	19 (29%)
Yes	-	-	46 (71%)
Unknown	-	-	15

* GI-GIV—Fuhrman Grade.

**Table 2:** The LOD and LOQ for different materials.

Analyte	Matrix	LOD (µg/L)	LOQ (µg/L)
^78^Se	Serum	0.0284	0.0862
^78^Se	Blood	0.0388	0.1295
^66^Zn	Serum	0.4953	1.4859
^66^Zn	Blood	0.5458	1.7861

**Table 3:** Comparison of obtained and certified values of concentrations of selenium and zinc.

Analyte	Material	Certified Value (µg/L)	Obtained Value (µg/L)
^78^Se	Serum (Plasmonorm L1)	66.1 ± 13.2	65.01
^78^Se	Serum (Sero L1)	95 ± 19	89.10
^78^Se	Blood (Plasmonorm L1)	75.3 ± 15	68.80
^66^Zn	Serum (Plasmonorm L1)	1320 ± 200	1286.40
^66^Zn	Serum (Sero L1)	1460 ± 290	1301.92
^66^Zn	Blood (Plasmonorm L1)	4580 ± 910	4480

**Table 4:** Correlation between Se levels in blood and deaths of kidney cancer patients.

	Vital Status			Univariable Cox Regression			Multivariable Cox Regression		
Variables	Overall N = 284 ^1^	Alive N = 204 ^1^	Deceased N = 80 ^1^	HR ^2^	95% CI ^2^	*p*- Value	HR ^2^	95% CI ^2^	*p*- Value
Se									
IV (reference) 109.35–200.03 (123.28)	71 (25%)	66 (32%)	5 (6.3%)	—	—		—	—	
I 13.50–86.24 (75.07)	71 (25%)	35 (17%)	36 (45%)	10.3	4.03, 26.2	<0.001	7.74	2.98, 20.1	<0.001
II 86.45–98.10 (91.78)	71 (25%)	50 (25%)	21 (26%)	4.86	1.83, 12.9	0.001	3.92	1.45, 10.6	0.007
III 98.10–108.93 (102.92)	71 (25%)	53 (26%)	18 (23%)	4.14	1.54, 11.2	0.005	3.85	1.41, 10.5	0.008

^1^ n (%), ^2^ HR = Hazard Ratio, CI = Confidence Interval.

**Table 5:** Correlation between Se levels in serum and deaths of kidney cancer patients.

	Vital Status			Univariable COX Regression			Multivariable COX Regression		
Variables	Overall N = 284 ^1^	Alive N = 204 ^1^	Deceased N = 80 ^1^	HR ^2^	95% CI^2^	*p-* Value	HR ^2^	95% CI ^2^	*p-* Value
Se									
IV (reference) 87.20–160.31 (97.72)	71 (25%)	64 (31%)	7 (8.8%)	—	—		—	—	
I 0.57–65.66 (57.68)	71 (25%)	38 (19%)	33 (41%)	5.76	2.55, 13.0	<0.001	3.97	1.71, 9.21	0.001
II 65.70–76.55 (71.01)	71 (25%)	44 (22%)	27 (34%)	4.35	1.89, 10.0	<0.001	3.54	1.52, 8.25	0.003
III 76.58–86.66 (80.85)	71 (25%)	58 (28%)	13 (16%)	1.92	0.76, 4.80	0.2	1.80	0.71, 4.54	0.2

^1^ n (%), ^2^ HR = Hazard Ratio, CI = Confidence Interval.

## Data Availability

Our data contains potentially sensitive information; therefore, we have not included it in our manuscript. Those who would like to request access to our data may contact Melissa Sidhu at the Research Ethics Board of Women’s College Hospital by calling (416) 351-3732 x2723 or email ac.latipsohcw@uhdis.assilem. The Pomeranian University of Medicine Ethics Committee will grant all researchers who meet the criteria access to confidential data. Availability of data and materials are included in the manuscript.

## Supplementary Materials

The following supporting information can be downloaded at https://www.mdpi.com/article/10.3390/biomedicines12081775/s1, Table S1. Survival from kidney cancer depending on blood Se levels in men; Table S2. Survival from kidney cancer depending on blood Se levels in women; Table S3. Survival from kidney cancer depending on blood Zn/Se ratio in men; Table S4. Survival from kidney cancer depending on blood SeQI-ZnQI vs. SeQIV-ZnQIV; Table S5. Survival from kidney cancer depending on serum Se levels in men; Table S6. Survival from kidney cancer depending on serum Se levels in women; Table S7. Survival from kidney cancer depending on serum Zn/Se ratio in men; Table S8. Survival from kidney cancer depending on serum SeQI-ZnQI vs. SeQIV-ZnQIV; Table S9. Zn correlation between levels in blood and deaths of kidney cancer patients; Table S10. Zn correlation between levels in serum and deaths of kidney cancer patients.
