# Supplementary material for: Blood and Serum Se and Zn Levels and 10-Year Survival of Patients after a Diagnosis of Kidney Cancer
Source: Biomedicines. 2024 Aug 6;12(8):1775. doi: 10.3390/biomedicines12081775 (PMC11351416; doi:10.3390/biomedicines12081775)
Supplement: Supplementary file 1 [file biomedicines-12-01775-s001.zip › biomedicines-3094677-supplementary.pdf]

Supplementary materials

Table S1. Survival from kidney cancer depending on blood Se levels in men

	Vital status			Univariable COX Regresion			Multivariable COX Regression		
**Variables**	**Overall, N=166[1]**	**Alive N=113[1]**	**Deceased N=53[1]**	**HR[2]**	**95% CI[2]**	***p*-value**	**HR[2]**	**95% CI[2]**	***p*-value**
Se									
IV (reference) 111.17 - 200.03 (126.53)	42 (25%)	39 (35%)	3 (5.7%)	—	—		—	—	
I 13.50 - 85.11 (74.04)	42 (25%)	14 (12%)	28 (53%)	17.4	5.14, 58.7	<0.001	11.6	3.31, 40.4	<0.001
II 85.85 - 98.71 (92.29)	41 (25%)	28 (25%)	13 (25%)	5.56	1.57, 19.6	0.008	4.43	1.24, 15.9	0.022
III 98.84 - 110.65 (103.85)	41 (25%)	32 (28%)	9 (17%)	4.03	1.07, 15.2	0.040	3.07	0.80, 11.8	0.10

[1]n (%),[2]HR = Hazard Ratio, CI = Confidence Interval

,

Table S2. Survival from kidney cancer depending on blood Se levels in women

	Vital status			Univariable COX Regresion			Multivariable COX Regression		
**Variables**	**Overall, N=118**[1]	**Alive N=91**[1]	**Deceased N=27**[1]	**HR**[2]	**95% CI**[2]	***p*-value**	**HR**[2]	**95% CI**[2]	***p*-value**
Se									
IV (reference) 106.64 - 163.14 (117.84)	30 (25%)	26 (29%)	4 (15%)	—	—		—	—	
I 41.92 - 87.09 (76.94)	30 (25%)	23 (25%)	7 (26%)	1.93	0.56, 6.59	0.3	1.52	0.43, 5.35	0.5
II 87.44 - 96.80 (91.36)	29 (25%)	20 (22%)	9 (33%)	2.59	0.80, 8.43	0.11	1.03	0.26, 4.01	>0.9
III 96.95 - 106.54 (101.65)	29 (25%)	22 (24%)	7 (26%)	1.90	0.56, 6.48	0.3	1.39	0.39, 5.03	0.6

[1]n (%),[2]HR = Hazard Ratio, CI = Confidence Interval

Table S3. Survival from kidney cancer depending on blood Zn/Se ratio in men

	Vital status			Univariable COX Regression			Multivariable COX Regression		
**Variables**	**Overall, N=166**[1]	**Alive N=113**[1]	**Deceased N=53**[1]	**HR**[2]	**95% CI**[2]	***p*-value**	**HR**[2]	**95% CI**[2]	***p*-value**
Zn/Se									
I(reference) 0.05 - 56.71	42 (25%)	34 (30%)	8 (15%)	—	—		—	—	
II: 56.74 - 66.27	41 (25%)	34 (30%)	7 (13%)	0.96	0.34, 2.66	>0.9	0.60	0.21, 1.74	0.3
III: 66.49 - 76.32	41 (25%)	28 (25%)	13 (25%)	2.09	0.85, 5.15	0.11	1.53	0.59, 3.98	0.4
IV: 76.59 - 99.25	42 (25%)	17 (15%)	25 (47%)	4.51	1.99, 10.3	<0.001	2.53	1.06, 6.03	0.037

[1]n (%),[2]HR = Hazard Ratio, CI = Confidence Interval

Table S4. Survival from kidney cancer depending on blood SeQI-ZnQI vs. SeQIV-ZnQIV

	Vital status			Univariable COX Regression			Multivariable COX Regression		
**Variables**	**Overl N=284[1]**	**Alive N=20[1]**	**Deceased N=80[1]**	**HR[2]**	**95% CI[2]**	***p*-value**	**HR[2]**	**95% CI[2]**	***p*-value**
SeQIZnQI vs. SeQIVZnQIV									
SeQ4ZnQ4 (reference): 109.35 - 200.03	23 (8.1%)	22 (11%)	1 (1.3%)	—	—		—	—	
Other: 63.51 - 169.82	229 (81%)	163 (80%)	66 (83%)	7.58	1.05, 54.6	0.044	7.64	1.05, 55.5	0.044
SeQ1ZnQ1_: 13.50 - 86.24	32 (11%)	19 (9.3%)	13 (16%)	12.1	1.58, 92.2	0.016	12.4	1.61, 96.2	0.016

[1]n (%),[2]HR = Hazard Ratio, CI = Confidence Interval

Table S5. Survival from kidney cancer depending on serum Se levels in men

	Vital status			Univariable COX Regresion			Multivariable COX Regression		
**Variables**	**Overall, N=166[1]**	**Alive N=113[1]**	**Deceased N=53[1]**	**HR[2]**	**95% CI[2]**	***p*-value**	**HR[2]**	**95% CI[2]**	***p*-value**
Se									
IV (reference) 87.89 - 150.77 (98.99)	42 (25%)	37 (33%)	5 (9.4%)	—	—		—	—	
I 35.06 - 65.81 (58.28)	42 (25%)	18 (16%)	24 (45%)	6.52	2.48, 17.1	<0.001	4.95	1.82, 13.5	0.002
II 65.89 - 77.35 (71.73)	41 (25%)	20 (18%)	21 (40%)	5.17	1.95, 13.7	<0.001	3.88	1.42, 10.6	0.008
III 77.53 - 87.66 (82.35)	41 (25%)	38 (34%)	3 (5.7%)	0.57	0.14, 2.38	0.4	0.49	0.12, 2.10	0.3

[1]n (%),[2]HR = Hazard Ratio, CI = Confidence Interval

Table S6. Survival from kidney cancer depending on serum Se levels in women

	Vital status			Univariable COX Regresion			Multivariable COX Regression		
**Variables**	**Overall, N=118**[1]	**Alive N=91**[1]	**Deceased N=27**[1]	**HR**[2]	**95% CI**[2]	***p*-value**	**HR**[2]	**95% CI**[2]	***p*-value**
Se									
IV (reference) 82.75 - 160.31 (94.81)	30 (25%)	25 (27%)	5 (19%)	—	—		—	—	
I 0.57 - 65.61 (57.12)	30 (25%)	21 (23%)	9 (33%)	1.87	0.62, 5.57	0.3	0.71	0.21, 2.36	0.6
II 65.66 - 76.33 (70.22)	29 (25%)	22 (24%)	7 (26%)	1.52	0.48, 4.78	0.5	1.20	0.34, 4.27	0.8
III 76.35 - 82.55 (79.24)	29 (25%)	23 (25%)	6 (22%)	1.26	0.39, 4.14	0.7	0.88	0.24, 3.18	0.8

[1]n (%),[2]HR = Hazard Ratio, CI = Confidence Interval

Table S7. Survival from kidney cancer depending on serum Zn/Se ratio in men

	Frequency of deaths			Univariable COX Regression			Multivariable COX Regression		
**Variables**	**Overall, N=166[1]**	**Alive N=113[1]**	**Deceased N=53**	**HR[2]**	**95% CI[2]**	***p*-value**	**HR[2]**	**95% CI[2]**	***p*-value**
Zn/Se_									
I(reference): 5.75 - 10.08	42 (25%)	36 (32%)	6 (11%)	—	—		—	—	
II: 10.12 - 11.31	41 (25%)	29 (26%)	12 (23%)	2.07	0.78, 5.54	0.15	1.65	0.61, 4.48	0.3
III: 11.34 - 13.07	41 (25%)	28 (25%)	13 (25%)	2.54	0.97, 6.70	0.059	3.32	1.24, 8.91	0.017
IV: 13.08 - 23.30	42 (25%)	20 (18%)	22 (42%)	4.08	1.65, 10.1	0.002	3.95	1.57, 9.98	0.004

[1]n (%),[2]HR = Hazard Ratio, CI = Confidence Interval

Table S8. Survival from kidney cancer depending on serum SeQI-ZnQI vs. SeQIV-ZnQIV

	Vital status			Univariable COX Regression			Multivariable COX Regression		
**Variables**	**Overall N=284[1]**	**Alive N=204[1]**	**Deceased N=80[1]**	**HR[2]**	**95% CI[2]**	***p*-value**	**HR[2]**	**95% CI[2]**	***p*-value**
SeQIZnQI vs. SeQIVZnQIV									
SeQ4ZnQ4 (reference): 87.20 - 160.31	28 (9.9%)	24 (12%)	4 (5.0%)	—	—		—	—	
Other: 42.53 - 150.77	222 (78%)	163 (80%)	59 (74%)	2.04	0.74, 5.61	0.2	1.82	0.65, 5.10	0.3
SeQ1ZnQ1 0.57 - 65.66	34 (12%)	17 (8.3%)	17 (21%)	4.56	1.53, 13.6	0.006	3.11	1.01, 9.63	0.049

[1]n (%),[2]HR = Hazard Ratio, CI = Confidence Interval

Table S9. Zn correlation between levels in blood and deaths of kidney cancer patients.

	Vital status			Univariable COX Regresion			Multivariable COX Regression		
**Variables**	**Overall, N=284[1]**	**Alive N=204[1]**	**Deceased N=80[1]**	**HR[2]**	**95% CI[2]**	***p*-value**	**HR[2]**	**95% CI[2]**	***p*-value**
Zn									
IV(reference): 6,946.63 - 9,206.50	71 (25%)	48 (24%)	23 (29%)	—	—		—	—	
I: 0.68 - 5,708.67	71 (25%)	46 (23%)	25 (31%)	1.12	0.63, 1.97	0.7	1.29	0.71, 2.36	0.4
II: 5,724.69 - 6,374.45	71 (25%)	51 (25%)	20 (25%)	0.83	0.45, 1.50	0.5	0.95	0.51, 1.77	0.9
III: 6,375.10 - 6,943.56	71 (25%)	59 (29%)	12 (15%)	0.46	0.23, 0.93	0.030	0.52	0.26, 1.06	0.072

[1]n (%),[2]HR = Hazard Ratio, CI = Confidence Interval

Table S10. Zn correlation between levels in serum and deaths of kidney cancer patients.

	Vital status			Univariable Cox Regresion			Multivariable Cox Regression		
**Variables**	**Overall N=284[1]**	**Alive N=204[1]**	**Deceased N=80[1]**	**HR[2]**	**95% CI[2]**	***p*-value**	**HR[2]**	**95% CI[2]**	***p*-value**
Zn									
IV(reference) 951.99 - 1,280.44	71 (25%)	57 (28%)	14 (18%)	—	—		—	—	
I: 0.36 - 755.99	71 (25%)	41 (20%)	30 (38%)	2.64	1.40, 4.98	0.003	1.92	0.98, 3.75	0.055
II: 756.27 - 847.72	71 (25%)	53 (26%)	18 (23%)	1.38	0.69, 2.78	0.4	1.30	0.62, 2.70	0.5
III: 848.02 - 948.98	71 (25%)	53 (26%)	18 (23%)	1.37	0.68, 2.75	0.4	1.13	0.55, 2.32	0.7

[1]n (%),[2]HR = Hazard Ratio, CI = Confidence Interval
